# Examination of the Accuracy of Movement Tracking Systems for Monitoring Exercise for Musculoskeletal Rehabilitation

**DOI:** 10.3390/s23198058

**Published:** 2023-09-24

**Authors:** Artem Obukhov, Andrey Volkov, Alexander Pchelintsev, Alexandra Nazarova, Daniil Teselkin, Ekaterina Surkova, Ivan Fedorchuk

**Affiliations:** 1 Laboratory of VR Simulators, Tambov State Technical University, 392000 Tambov, Russia; didim@eclabs.ru (A.V.); nazarova.al.ol@yandex.ru (A.N.); dteselk@mail.ru (D.T.); esur2506@yandex.ru (E.S.); ivan.fedorchuk.42@gmail.com (I.F.); 2 Department of Higher Mathematics, Tambov State Technical University, 392000 Tambov, Russia; pchelintsev.an@yandex.ru

**Keywords:** musculoskeletal rehabilitation, motion tracking systems, machine learning, human positioning accuracy

## Abstract

When patients perform musculoskeletal rehabilitation exercises, it is of great importance to observe the correctness of their performance. The aim of this study is to increase the accuracy of recognizing human movements during exercise. The process of monitoring and evaluating musculoskeletal rehabilitation exercises was modeled using various tracking systems, and the necessary algorithms for processing information for each of the tracking systems were formalized. An approach to classifying exercises using machine learning methods is presented. Experimental studies were conducted to identify the most accurate tracking systems (virtual reality trackers, motion capture, and computer vision). A comparison of machine learning models is carried out to solve the problem of classifying musculoskeletal rehabilitation exercises, and 96% accuracy is obtained when using multilayer dense neural networks. With the use of computer vision technologies and the processing of a full set of body points, the accuracy of classification achieved is 100%. The hypotheses on the ranking of tracking systems based on the accuracy of positioning of human target points, the presence of restrictions on application in the field of musculoskeletal rehabilitation, and the potential to classify exercises are fully confirmed.

## 1. Introduction

Diseases of the musculoskeletal system are widespread among different age groups and population categories. Trauma or illness can affect both the physical function and the cognitive and emotional state of a person. Studies show that people in need of rehabilitation often experience helplessness, frustration, and social isolation, which are associated with increased depression and a decreased ability to perform their daily activities [1,2,3]. The incomplete list of possible deficiencies includes muscle weakness, poor endurance, a lack of muscle control, spatial neglect, and even paralysis. Delayed or insufficient motor rehabilitation leads to the worsening of the patient’s condition. Thus, a person becomes dependent on the regular assistance of others.

Exercise-based therapy in combination with other methods of musculoskeletal rehabilitation is a logical approach to restoring and strengthening motor abilities in patients at risk of progressive musculoskeletal dysfunction. It is proven that with the systematic execution of time-consuming and complex therapeutic exercises, as a rule, there is a better functional recovery [4,5,6]. Therefore, hospital rehabilitation of the patient is an integral stage of treatment. The term “rehabilitation” refers to the complete or partial compensation of physical abilities through physical exercises for the recovery of a person in social, domestic, and professional environments.

The advantage of hospital rehabilitation compared to ambulatory rehabilitation is a question to be discussed. There are studies that confirm the fact that rehabilitation under the supervision of a specialist does not exceed rehabilitation at home [7,8,9,10] and vice versa [11,12,13,14].

As part of this study, it is assumed that the patient should receive primary controlled rehabilitation and then continue to follow the doctor’s prescriptions independently. This is necessary because not all patients can afford a permanent residence in a medical institution for the following reasons: insufficient hospital beds, high healthcare costs, a shortage of medical personnel, and the prevention of intra-hospital infections. Ambulatory rehabilitation relieves the healthcare burden and allows patients to continue their social, household, and professional activities throughout the recovery period.

Studies show that modern information technologies can improve the level of information provided to patients and their relatives about the progress of ambulatory rehabilitation [15] and that personalized medicine can lead to improved life quality for patients. In addition, mobile apps can increase the availability of rehabilitation in both rural and urban environments, reduce travel time and costs, increase involvement in therapeutic activities, and ensure decision making with the doctor regarding the treatment process. Smart wearable devices, combined with software that supports advanced analytics, can provide the user with the ability to track the dynamics of the rehabilitation process at home without constant medical supervision.

Thus, the contribution of this study is as follows:

- Analysis of existing systems for tracking human movements and their applicability in musculoskeletal rehabilitation;
- Modeling the process of monitoring and evaluating musculoskeletal rehabilitation exercises using various tracking systems, which can be used in the development of rehabilitation systems of various kinds;
- Development and testing of information-processing algorithms for various systems of movement tracking to obtain objective data on the quality of the exercise performance;
- Implementation of algorithms for the classification of human actions to automatically determine the exercise performed.

The content of this article includes an introduction that analyzes existing systems for tracking movements, approaches to data analysis on the process of performing musculoskeletal rehabilitation exercises, and methods of classification using machine learning. The following is a model of the process of monitoring and evaluating musculoskeletal rehabilitation exercises, as well as the necessary algorithms of data processing and analysis used to obtain objective indicators of exercise performance quality and recognition of actions. Section 3 provides details of the experimental studies that include comparing the accuracy of different human movement tracking systems, choosing the optimal solution for the subject area of musculoskeletal rehabilitation, and comparing machine learning algorithms for automatic classification of exercises. This article ends with a discussion of the results and conclusions.

### 1.1. Monitoring of User Movements

One of the components of the rehabilitation dynamics evaluation process is the analysis of the person’s motor activity, for which it is necessary to obtain not only a qualitative assessment from a specialist but also objective quantitative data. Next, the main directions and systems that allow tracking of user movements are considered.

#### 1.1.1. Inertial Navigation Systems

Inertial navigation systems (INS) contain linear acceleration sensors (accelerometers) measuring non-gravitational acceleration on three axes (*x*, *y*, and *z*) and angular speed (gyroscopes or pairs of accelerometers measuring radial acceleration), determining deviation and angles of orientation.

The use of accelerometers in collecting the data on movement provides quantitative measurements and allows for specific changes in behavior when rehabilitating people. Moreover, these devices are used to objectively quantify the level of ambulatory activity of patients [16]. When evaluating human movements, an important component is the orientation in space, which cannot be determined only by using accelerometers because they do not contain information about rotation around the vertical axis. The combination of accelerometers with gyroscopes allows for a more comprehensive analysis of motion [17,18].

Combinations of accelerometers and gyroscopes are actively used to assess human motor activity during the rehabilitation period [19]. It should be noted that current research in this direction is based on the use of specialized accelerometers and gyroscopes presented as separate devices, which limits the application of these developments to a broad audience. This is due to the fact that the data provided by mobile accelerometers are not always of high accuracy and quality. This may require the development of new approaches to information processing to address the high error rate of mobile devices.

#### 1.1.2. Steam VR Lighthouse

A number of virtual reality systems include support for wireless sensors implemented in the form of controllers, trackers, or other manipulators running on Steam VR Lighthouse technology (e.g., HTC Vive) [20]. For these systems, open-source software libraries (OpenVR) were implemented, which allowed the systems to obtain sensor location data with high frequency and accuracy without additional calculations. Alternative solutions from Oculus, Valve, and HTC, built on computer vision and a set of cameras for monitoring controllers, do not allow monitoring of all target body points except hands and fingers, which makes such solutions inapplicable for this study.

There is a positive experience with using this kind of tracking system in the field of rehabilitation [21]; however, to improve tracking accuracy, the authors combined virtual reality equipment with computer vision technologies.

The disadvantage of Steam VR Lighthouse is the need to organize a tracking area with the installation and configuration of base stations. The tracker coordinates depend on the initial calibration of the virtual reality headset, and the sensors must be located in the visibility zone of the base stations, which often leads to data loss in the event of their cover with the user’s body or clothes.

The experience of using such sensors also shows that the Steam VR Lighthouse-based tracking system does not accommodate a large number of trackers due to their size, and the scan area of base stations is limited.

#### 1.1.3. Motion Capture Suit

Motion capture suits are implemented on the basis of a system of special markers, sensors, or trackers located on the torso, hands, and legs, combined into a single system [22,23]. Such systems allow accurate recording of the entire body’s movements, including wirelessly. The disadvantages of the approach are the need for calibration as well as the low interference resistance, which makes it difficult to use such equipment near sources of electromagnetic interference.

Another peculiarity of the suits is that the recording of data is carried out in relation to some basic supporting point, usually fixed on the back. The sensor installed at this point records the shifts and twists on three axes, while the other sensors installed on the target positions of the human body record the change in the angles of twist relative to the previous position.

Since the lengths of segments between the sensors are fixed, the recorded and digitized model of human movement does not reflect the user’s height or the length of their limbs. This requires additional data processing taking into account the height of the current user to determine the actual location of the points of the digital model. Therefore, when using motion capture suits in various three-dimensional application development environments (Unity, Unreal Engine, and others), body sizes are set by the developer manually. This feature can be used when evaluating the quality of the performance of musculoskeletal rehabilitation exercises, when the length of the limbs and height are determined for each user, after which these values are transferred to a digital model. Then, the resulting model and the characteristics of its movement correspond to the real processes of movement.

#### 1.1.4. Computer Vision Systems

Tracking systems, based on the use of cameras or some of their sets with the subsequent application of computer vision and machine learning technologies, allow the capture of movements of the human body, including hands and face. Such technologies, for example, are implemented in libraries like MediaPipe [24], MoveNet [25], and OpenPose [26,27]. These libraries allow developers to cover a sufficient area of the image and track several people simultaneously with a large number of points (up to 500 points, including fingers and faces).

Practical tests show that with sharp movements or large distance from the camera, the accuracy of segment recognition significantly decreases, and erroneous movements of the recognized skeleton nodes appear. This approach also demonstrates low performance due to the use of algorithms with high computation complexity based on neural networks. A significant limitation is also that the use of a single camera does not allow the correct determination of the third coordinate of the points (by the *Z* axis), that is, the distance from the camera to the object. In a number of musculoskeletal rehabilitation exercises where it is necessary to make simple movements on one or two axes, the impact of these shortcomings is reduced. On the other hand, the use of a set of cameras that analyze the object from different sides, specialized cameras with a depth sensor, or stereo cameras also allows one to obtain the necessary data on the human body segments’ position.

### 1.2. Overview of Approaches to the Analysis and Classification of User Movements in the Process of Musculoskeletal Rehabilitation

In the process of musculoskeletal rehabilitation, a person performs a sufficiently large number of various exercises aimed at working out different muscle groups. Automatic recognition of the exercise performed allows one more accurately to determine the quality of its performance, since certain limit conditions can be set for each exercise to which a person should strive in the process of rehabilitation.

An important tool for the automatic analysis and classification of user movements is the use of machine learning algorithms. We consider the specifics and experience of their application in this subject area.

The study [28] examined the use of LSTM networks trained on the wave conversion of EMG data in the rehabilitation process to assess the normality of the EMG response to rehabilitation actions. The authors achieved an average accuracy of more than 94%. A review of the application of different types of neural networks in the data classification for musculoskeletal medicine shows that these technologies can provide recognition accuracy of 70 to 95% [29].

The effectiveness of various machine learning algorithms (decision tree, k-nearest neighbor, support vector method, and random forests) in the classification of exercise for shoulder joint rehabilitation is also confirmed in the study [30], where the authors obtained accuracy of up to 97%. Classification models showed high efficiency in differentiating a patient’s physical activity and determining a specific type of exercise using inertial sensor data. Similar to the abovementioned study, the article [31] presented the results of machine learning algorithms (k-nearest neighbors, reference vector method) in managing the rehabilitation planning of elderly patients at home. The main purpose of using these algorithms is to identify the best method for predicting rehabilitation potential. Subjective user assessments of functional improvements in their state were used as input data. As a result, it was discovered that machine learning algorithms could be useful in developing improved clinical protocols.

The methodology developed by the authors of article [32] for modeling and evaluating human movements in the process of physical rehabilitation therapy is based on a combination of a sensory movement recording system and a trajectory extraction algorithm. After analyzing the trajectory and comparing it with the reference, a recommendation is issued for a further course of rehabilitation. The functioning of this algorithm is based on the use of a recurrent neural network that analyzes the spatial–temporal dependencies of the user’s movements to form a final assessment of the exercise performance.

A comprehensive overview of modern research in the field of human movement recognition is presented in the paper [33], which examines various tracking technologies (such as devices, smartphones, radar, and vision devices) together with different architectures of convolutional neural networks (with the addition of recurrent and generative models and layers of attention). There is a great prospect of combining machine learning technologies with tracking systems to solve various tasks in the healthcare, video surveillance, entertainment, and sports industries.

In particular, an overview of scientific papers exploring the assessment of human movement through vision devices is also presented in the article [34]. Modern researchers in the field of optical motion capture are briefly described in relation to a number of parameters, as well as sets of open-source software tools, such as PoseNet [35] and OpenPose [36], mainly based on algorithms of convolutional neural networks [37,38,39].

In recognizing human movements, researchers use various machine learning architectures, including increasingly popular attention-based models (Transformers). In the study [40], accuracy in recognizing human actions reached 99.2%.

Thus, in the course of studying existing research in the field of the machine learning algorithms application for movement classification, the following machine learning algorithms were selected due to their effectiveness for solving similar tasks:

- Solution Trees: A simple-to-implement and easily interpreted machine learning algorithm that identifies the characteristics by which the classification was completed; the greater depth of the tree, the more branches by different characteristics are performed for classification;
- K-nearest neighbors: The classification is performed on the basis of comparing the current object characteristics with the parameters of the nearest objects and choosing a similar class to them;
- Random forest: an ensemble method of classification combining several assessors (trees of solutions with a given depth of branching) to increase the accuracy;
- Multilayered dense neural networks: simple to implement; universal approximators;
- Recurrent neural networks such as LSTM: common and effective models in time series analysis;
- Multilayered convolutional neural networks: generalize the signs of time sequences and rows, which is justified for most types of data coming from tracking systems;
- Multilayered convolutional neural networks with the addition of layers of multiheaded attention (Transformer architecture): an efficient and modern type of neuronal network that includes, in addition to convolutional neural networks, layers of multiheaded attention, allowing one to identify specific features in incoming datasets.

As a result of the analysis, the applicability of machine learning algorithms in solving tasks of analysis and classification of movements in the process of rehabilitation exercises is confirmed. As part of this study, these techniques are applied to classify selected types of exercises.

### 1.3. Analysis of Approaches to Assessing User Tracking Quality

In the process of performing musculoskeletal rehabilitation exercises, an important component of monitoring and evaluating this process is the precise determination of the position of the human body target points. We consider the main approaches and metrics that can be used in the process of evaluating user tracking quality.

Dynamic time warping (*DTW*) is one of the most commonly used algorithms for finding similarities between time series. Its purpose is to find the optimal global alignment between two time series using time distortions.

According to the study [41], if two time series (*T*_1_ and *T*_2_) are set with the number of measurements at coordinates $P_{1}$ and $P_{2}$, the *DTW* algorithm builds a matrix of distances between the corresponding elements of two trajectories of the size of $P_{1}\times P_{2}$. Further, in this matrix, a certain path of transformation $W=\{\omega_{k}\}$ by length K is determined, establishing a correspondence between the two trajectories. Then, if $d(\omega_{k})$ is the distance of some elements of two trajectories entering the path of transformation, then the *DTW* distance (path value) between them is calculated on the basis of the optimal path of transformation using the formula

(1) $$DTW(T_{1},T_{2})=\min\left[\frac{\sum_{k=1}^{K}d(\omega_{k})}{K}\right].$$

*DTW* works well when finding similarities between two trajectories if they are similar, but the main disadvantage of this algorithm is that it is noise sensitive, i.e., it gives meaningless results when comparing two trajectories containing many dissimilar sections. This can make it difficult to use this algorithm in the analysis of motor activity.

When analyzing the value of the detachment of the human body target point from some benchmark, the following common metrics can be used: the mean square error (*MSE*) and the Euclidean distance (*D*) between the points. On the basis of the last metrics for the trajectory of body movement, four different estimates can be given: the average, maximum, and total Euclidean distances between the current and reference trajectories of target point motion. For the calculation of the listed metrics, the following formulas are used when comparing the points of the current $T_{c}$ and the reference $T_{e}$ trajectory of human movement [42]:

*MSE*:

(2) $$MSE(T_{c},T_{e})=\frac{1}{N}\sum_{i=1}^{N}{\left(x_{c,i}-x_{e,i}\right)}^{2}+{\left(y_{c,i}-y_{e,i}\right)}^{2}+{\left(z_{c,i}-z_{e,i}\right)}^{2},$$

mean Euclidean distance:

(3) $$D_{mean}=\frac{1}{N}\sum_{j=1}^{N}\sqrt{{\left(x_{c,i}-x_{e,i}\right)}^{2}+{\left(y_{c,i}-y_{e,i}\right)}^{2}+{\left(z_{c,i}-z_{e,i}\right)}^{2}},$$

maximum Euclidean distance:

(4) $$D_{\max}=\underset{i=1\ldots N}{\max}\left(\sqrt{{\left(x_{c,i}-x_{e,i}\right)}^{2}+{\left(y_{c,i}-y_{e,i}\right)}^{2}+{\left(z_{c,i}-z_{e,i}\right)}^{2}}\right),$$

total Euclidean distance:

(5) $$D_{sum}=\sum_{i=1}^{N}\sqrt{{\left(x_{c,i}-x_{e,i}\right)}^{2}+{\left(y_{c,i}-y_{e,i}\right)}^{2}+{\left(z_{c,i}-z_{e,i}\right)}^{2}},$$

where N is the number of trajectory points analyzed;

$x_{c,i},y_{c,i},z_{c,i}$: the target point of the current trajectory;

$x_{e,i},y_{e,i},z_{e,i}$: reference trajectory point.

*MSE* and metrics based on the Euclidian distance can be used both to determine the accuracy of tracking the target body points and to assess the performance of the exercise.

For example, instead of comparing all points of trajectories, it is possible to calculate the Euclidean distance D only between the most important points determining the quality of the exercise (maximum position, distance passed by the point, etc.).

### 1.4. Purpose of the Research

The purpose of the research is to increase the accuracy of recognizing human movements in the process of performing musculoskeletal rehabilitation exercises. To achieve this, the following tasks need to be completed:

1. Simulate the processes of monitoring and evaluating musculoskeletal rehabilitation exercises, which include the formalization of procedures for determining positions, amplitudes, and speeds of body parts of the user, as well as determining the current exercise and its performance quality.
2. Develop the necessary information processing algorithms from various movement tracking systems to obtain the final result in the form of a set of target points needed to evaluate the exercises.
3. Implement an algorithm for monitoring musculoskeletal rehabilitation exercises to analyze and classify user movements.
4. Implement various user tracking systems, taking into account the characteristics of the subject area and the limitations arising in the process of musculoskeletal rehabilitation.
5. Compare intelligent information processing algorithms, including machine learning methods, to automate the process of classifying musculoskeletal rehabilitation exercises with the chosen movement tracking method.

During the investigation, the following hypotheses should also be checked:

**Hypothesis** **1.** *The tracking systems considered can be ranked by accuracy. Accuracy refers to the deviation of the obtained metrics of the exercise (the minimum and maximum positions of the target point relative to the reference).*

**Hypothesis** **2.** *A number of tracking systems, without consideration of their accuracy, have restrictions on application in the field of musculoskeletal rehabilitation due to the complexity of their use in ambulatory conditions.*

**Hypothesis** **3.** *The performance of the exercises by the participants of the control group can be classified using machine learning algorithms, taking into account the choice of a tracking system that provides the necessary accuracy.*

## 2. Materials and Methods

### 2.1. Modeling the Process of Monitoring and Assessing Musculoskeletal Rehabilitation Exercises

The process of monitoring and evaluating exercise during musculoskeletal rehabilitation can be formalized and described, regardless of the movement tracking system used. This allows the model to be used further in other studies as a basis for creating monitoring and evaluation systems for exercises. To model the process of monitoring and evaluating musculoskeletal rehabilitation exercises, we use the set theory. In the future, during the practical implementation of the model in the form of software, the set theory will allow it to be used without additional transformations, moving from sets and operations to classes and methods implemented in the selected programming language.

The basis of the process being considered is the formation of a set of target points necessary to assess the quality of the exercises performed. In the first phase, the main components (characteristics) of the exercises were analyzed. Let E be a multitude of exercises, and $e_{k}\in E$ be a few exercises from this multitude. Let us denote the trajectory of target point movement that determines the characteristics of exercise $e_{k}$ as $TP_{k}$. This trajectory has corresponding unique parameters, including initial and final positions, and movement speed.

The target point $tp_{i}\in TP_{k}$ is defined as the set of values of coordinates on three axes:

(6) $$tp_{i}=\langle x_{i},y_{i},z_{i}\rangle ,$$

where $x_{i}\in X$, $y_{i}\in Y$, and $z_{i}\in Z$ are the coordinates of the target point on the axes *X*, *Y*, and *Z*, respectively. Then, the multitude $TP_{k}$ reflects the dynamics of the target point coordinates’ change in the process of exercise $e_{k}\in E$. The sequence of points $tp_{i}$ in the set $TP_{k}$ is ordered as they are received from the tracking system, starting from the first and ending with the last, which completes the exercise.

Each exercise $e_{k}\in E$ can be matched by a certain tuple of its parameters:

(7) $$e_{k}\to \langle f_{k}^{x},f_{k}^{y},f_{k}^{z},p_{k},l_{k}\rangle ,$$

where $f_{k}^{x},f_{k}^{y},f_{k}^{z}$ is the point of the human body on three axes, calculated on the basis of polynomials or splines, algorithms of linear regression, or other approaches that provide minimal deviation from the initial target points:

(8) $$\begin{array}{l} \sum_{i=0}^{N}{\left(f_{k}^{x}(i\cdot \Delta t)-x_{i}\right)}^{2}\to \min,\\ \sum_{i=0}^{N}{\left(f_{k}^{y}(i\cdot \Delta t)-y_{i}\right)}^{2}\to \min,\\ \sum_{i=0}^{N}{\left(f_{k}^{z}(i\cdot \Delta t)-z_{i}\right)}^{2}\to \min. \end{array}$$

$p_{k}=N\cdot \Delta t$: time interval of the exercise in seconds;

N: the number of measurements of the target point;

Δt: the interval in seconds between measurements;

$l_{k}$: the boundary values of spatial and space–time characteristics of the movement of the target point of the exercise:

(9) $$l_{k}\to \langle x_{k}^{\min},x_{k}^{\max},y_{k}^{\min},y_{k}^{\max},z_{k}^{\min},z_{k}^{\max},\bar{sx_{k}},\bar{sy_{k}},\bar{sz_{k}}\rangle ,$$

where $x_{k}^{\min}=\min(X)$ is the minimum value of the target point position on the *X* axis, similar to $y_{k}^{\min}$ and $z_{k}^{\min}$ for the *Y* and *Z* axes, respectively;

$x_{k}^{\max}=\max(X)$ is the maximum value of the target point position on the *X* axis, similar to $y_{k}^{\max}$ and $z_{k}^{\max}$ for the *Y* and *Z* axes, respectively;

$\bar{sx_{k}},\bar{sy_{k}},\bar{sz_{k}}$ are the average values of the target point speed along the *X*, *Y*, and *Z* axes.

Each exercise can be matched by its category $c_{q}\in C$, reflecting the specific actions and movements necessary for the qualitative performance of this exercise:

(10) $$e_{k}\to c_{q}.$$

After determining the main objects of the subject area and their properties, the task of assessing the exercise quality is considered.

Let a subset of exercises $E_{e}\subset E$ be given with reference to trajectories and characteristics of movements (received, for example, under the supervision of the doctor). If a new exercise $e_{m}\in E$ of category $c_{q}$, has entered the database, it is compared with the reference exercise $e_{k}\in E_{e}$ of the same category $c_{q}$ by the following formulas:

(11) $$\begin{array}{ll} F & =\sum_{i=0}^{N}{\left(f_{m}^{x}(i\cdot \bar{\Delta t})-f_{k}^{x}(i\cdot \bar{\Delta t})\right)}^{2}+\sum_{i=0}^{N}{\left(f_{m}^{y}(i\cdot \bar{\Delta t})-f_{k}^{y}(i\cdot \bar{\Delta t})\right)}^{2}\\ & \;+\sum_{i=0}^{N}{\left(f_{m}^{z}(i\cdot \bar{\Delta t})-f_{k}^{z}(i\cdot \bar{\Delta t})\right)}^{2}, \end{array}$$

(12) $$P=\left\{ \begin{array}{l} 1,\;\mathrm{if}\;\left(p_{k}>p_{m}\right),\;\\ 0.5,\;\mathrm{if}\;\left(p_{k}<p_{m}\right),\\ 0,\;\mathrm{if}\;\left(p_{k}+\lambda p_{k}<p_{m}\right), \end{array}\right.$$

(13) $$\begin{array}{c} L_{D}=\sqrt{{\left(x_{m}^{\max}-x_{k}^{\max}\right)}^{2}+{\left(y_{m}^{\max}-y_{k}^{\max}\right)}^{2}+{\left(z_{m}^{\max}-z_{k}^{\max}\right)}^{2}}\\ \;+\sqrt{{\left(x_{m}^{\min}-x_{k}^{\min}\right)}^{2}+{\left(y_{m}^{\min}-y_{k}^{\min}\right)}^{2}+{\left(z_{m}^{\min}-z_{k}^{\min}\right)}^{2}}, \end{array}$$

(14) $$L_{S}=\frac{\bar{sx_{m}}+\bar{sy_{m}}+\bar{sz_{m}}}{\bar{sx_{k}}+\bar{sy_{k}}+\bar{sz_{k}}},$$

where F is the average standard deviation of the reference trajectory from the estimated;

$\bar{\Delta t}$: the arithmetic mean of time intervals between measurements of data, in seconds;

P: estimate the performance time of the exercise relative to the reference;

λ: the correctional coefficient of the exercise performance time, allowing one to estimate the excess of the reference time as a satisfactory result, is selected experimentally depending on the exercise (0<λ≤0.5);

$L_{D}$: estimate of the Euclidean distance between the minimum and maximum values of the target point in the current and reference exercises;

$L_{S}$: the assessment of the difference between the space–time characteristics (mean speed of movement) of the evaluated exercise and the reference exercise [43].

The maximum quality of the evaluated exercise is achieved when the following conditions are met:

(15) $$\begin{array}{c} F\to 0,\\ P=1,\\ L_{D}\to 0,\\ L_{S}\to 1. \end{array}$$

Thus, the evaluation of the exercise is calculated on the basis of the deviation from the values of the recorded reference exercise, or, in its absence, the threshold values are used for all metrics: the ideal trajectory for which the maximum and minimum positions of the target point are set, the recommended average speed, and the time of execution.

An important component of the process of monitoring and evaluating the exercises performed is the automatic determination of the exercise type (category). Two options are possible in the practical implementation of such systems: manual selection of exercises or automatic recognition. The approach described below can be used to automatically determine the category of exercise.

It is necessary to approximate the relationship between the trajectories of the target point and the exercise category using a machine learning method:

(16) ML(TP)=C.

Thus, ML displays multiple trajectories of target point movement in multiple categories of exercises. In addition to neural networks, other methods discussed in Section 1.2 may be used. Their effectiveness is evaluated further below.

For the presented model, a modification can be made in the case when not only one target point is tracked as part of the exercise but several. The set of $TP_{k}$ exercises $e_{k}$ in the tracking of m target points take the following form:

(17) $$TP_{k}=\left\{TP_{k,m}\right\},TP_{k,m}=\left\{tp_{i,m}\right\}$$

That is, m subsets are formed that store the trajectories of each of the m points. In Formula (6) and in the formulas below, it is necessary to make separate calculations for each of the target points, which can be averaged. Also, in Formula (16), not one trajectory is analyzed but a set of trajectories from all target points.

### 2.2. Data Processing Algorithms from Various Movement Tracking Systems for Exercise Monitoring

The model presented above in a generalized form formalizes the processes of monitoring and evaluating musculoskeletal rehabilitation exercises. For the successful application of this model, it is necessary to prepare the source data obtained from the movement tracking system and make them uniform so that the model can process them. We consider the appropriate algorithms for each tracking system.

#### 2.2.1. Processing Data from Inertial Navigation Systems

A distinctive feature of the INS, based on the calculation of indications from the accelerometer and the gyroscope, is the need to integrate accelerations on three axes, taking into account angles of turning and the impact of the geomagnetic field. All this leads to a huge error in determining the speed and trajectory of the target point movement. The following is a description of the necessary data conversions.

INS form the output data in the acceleration tuple $a_{i}$ of the target point on three axes:

(18) $$a_{i}=\langle ax_{i},ay_{i},az_{i}\rangle ,$$

where $ax_{i}\in AX$, $ay_{i}\in AY$, and $az_{i}\in AZ$ are accelerations along the *X*, *Y*, and *Z* axes, respectively, and AX, AY, and AZ are the sets of acceleration values along the corresponding axes. Note that when forming the tuple $a_{i}$ it is necessary to record data from the device’s gyroscope to take into account the angle of inclination of the device in space.

Each i-th data measurement is carried out after a period of time Δt. At the next step, the speed of movement of the device $s_{i}$ is determined:

(19) $$s_{i}=\langle \begin{array}{l} sx_{i}=sx_{i-1}+ax_{i}\cdot \Delta t,\\ sy_{i}=sy_{i-1}+ay_{i}\cdot \Delta t,\\ sz_{i}=sz_{i-1}+az_{i}\cdot \Delta t \end{array}\rangle ,$$

where $sx_{i}\in SX$, $sy_{i}\in SY$, and $sz_{i}\in SY$ are the velocities along the *X*, *Y*, and *Z* axes, respectively, and the sets SX, SY and SZ are the sets of velocity values along the corresponding axes.

The next step is to obtain increments of the target point trajectories along the three axes. Initially, the variables $x_{i}\in X$, $y_{i}\in Y$, and $z_{i}\in Z$ have zero values (when i=0). X, Y, and Z are the sets of values of the points of the target point trajectory along the corresponding axes. At each step with a time interval Δt, the obtained metrics are

(20) $$\begin{array}{c} x_{i}=x_{i-1}+sx_{i}\cdot \Delta t+\frac{ax_{i}\cdot \Delta t^{2}}{2},\\ y_{i}=y_{i-1}+sy_{i}\cdot \Delta t+\frac{ay_{i}\cdot \Delta t^{2}}{2},\\ z_{i}=z_{i-1}+sz_{i}\cdot \Delta t+\frac{az_{i}\cdot \Delta t^{2}}{2}. \end{array}$$

Thus, the tuple $tr_{i}=\langle x_{i},y_{i},z_{i}\rangle$ uniquely determines the position of the target point at time i⋅Δt from the start of the record. The set of target points $tr_{i}$ can then be used in the calculations of the model in Section 2.1 since its form and content correspond to the format given in (6). Therefore, all calculation formulas in Section 2.1 are applicable.

Due to the high measurement error, after integrating the initial data and accumulating the error, it may be necessary to apply filtering or data processing algorithms. That is, it is necessary to carry out a certain set of transformations of the FP of the initial data of the $a_{i}$ inertial navigation system in such a way that the average Euclidean distance D of the deviation of the processed trajectory of the target point from the real trajectory is minimal:

(21) $$\begin{array}{c} D_{mean}(FP)=\frac{1}{N}\sum_{i=1}^{N}\sqrt{{\left(x_{i}^{*}-FP(ax_{i})\right)}^{2}+{\left(y_{i}^{*}-FP(ay_{i})\right)}^{2}+{\left(z_{i}^{*}-FP(az_{i})\right)}^{2}},\\ D_{mean}(FP)\to \min, \end{array}$$

where $FP(ax_{i})$, $FP(ay_{i})$, and $FP(az_{i})$ are the positions of the point along the *X*, *Y*, and *Z* axes, respectively, calculated on the basis of the inertial navigation system data using filtering and signal processing algorithms. FP, $x_{i}^{*}$, $y_{i}^{*}$, and $z_{i}^{*}$ are the values of the corresponding real coordinates of the target point.

The linear Kalman filter [44] implemented in the FilterPy library [45] is used as the main filter in this study. Using this filter, it is possible to carry out a relatively accurate calculation of the speed and trajectory [46,47] in accordance with Formulas (19) and (20), as well as to remove noise.

#### 2.2.2. Processing Data from Virtual Reality Systems

Virtual reality trackers and controllers powered by Steam VR Lighthouse technology provide coordinates from all sensors and angles of inclination with high frequency and precision. Thus, a data processing algorithm is not required for this class of systems, as the target point is originally formed in Formula (6). Velocity and acceleration can be obtained by differentiation.

When working with trackers or sensors of virtual reality systems, it is necessary to carry out initial calibration to obtain coordinates normalized relative to the initial position. This process does not cause difficulties, since it consists of saving the coordinate values of target points obtained during calibration and subtracting these values from the current ones, which can be integrated into the data acquisition system.

#### 2.2.3. Processing Data from Motion Capture Suit

As a result of the motion capture suit use, a set of one base point and a multitude of segments (bones) located in relation to it is formed, the position of which is indicated by angles of inclination on three axes. If necessary, the system allows one to record, in addition to changes in the sensor angle, its movement relative to the previous measurement [23].

Mark the base point on the back of the user as $b_{0}=\langle bx_{0},by_{0},bz_{0},bax_{0},bay_{0},baz_{0}\rangle$, the tuple contains coordinates on three axes and angles of turns. At a certain point in time j is given a number of segments (bones) $B_{j}=\{b_{i}|i=1\ldots N_{b}\}$, the total number of $N_{b}$. For each segment $b_{i}$ three values are given:

(22) $$b_{i}=\langle bax_{i},bay_{i},baz_{i}\rangle ,$$

where $bax_{i},bay_{i},baz_{i}$ are the characteristics of the turn of the i-th sensor on three axes, relative to the previous measurement.

A multitude of B segments is set for each measurement, thus forming a sequence of $\left\{B_{1},B_{2},\ldots ,B_{N}\right\}$, containing information about all the movements of the human body model. This sequence is transmitted to a development environment capable of processing recorded animations from motion capture suits.

The next stage of data processing is the selection of target points on the digital model of the human body [42]. To achieve this, it is necessary to set the size of the human body model (height and length of the limbs), after which a number of target points is set. Each target point $tp_{i}$ is attached to the nearest segment $b_{k}$ of the digital model (and the segment is higher in the skeletal model hierarchy than the target point):

(23) $$b_{k}\to tp_{i},$$

(24) $$tp_{i}=\langle bx_{k}+\Delta x_{i},by_{k}+\Delta y_{i},bz_{k}+\Delta z_{i}\rangle ,$$

where $bx_{k},by_{k},bz_{k}$ is the position of the starting point of the segment in the metric coordinates of the virtual scene, the scales of which are close to the real world;

$\Delta x_{i},\Delta y_{i},\Delta z_{i}$ is the distance between the start point of the segment and the selected target point.

Then, when the segment is shifted, taking into account the fixed distances $\Delta x_{i},\Delta y_{i},\Delta z_{i}$, changing the position of $bx_{k},by_{k},bz_{k}$ leads to obtain the current position of the target point. Thus, Formula (24) corresponds in form to (6) and can be used to assess the quality of the exercise.

#### 2.2.4. Processing Data in Computer Vision Systems

The data processing algorithm for tracking the human body by computer vision systems has certain similarities with the algorithm presented in Section 2.2.3 but has the following features:

- There is no reference point;
- All points of the human body model have their own coordinates;
- The coordinates of body model points are given in relative values (from 0 to 1), in accordance with the position on the frame received from the camera.

These features lead to the need to perform the following conversions on the source data. The first phase of the algorithm involves extracting from the frame $f_{j}$, obtained at time j, a set of points $P_{j}=\{p_{i}\}$:

(25) $$f_{j}\to P_{j},$$

(26) $$p_{i}=\langle px_{i},py_{i},pz_{i}\rangle .$$

where $px_{i},py_{i},pz_{i}$ are the coordinates of point $p_{i}$ in frame $f_{j}$ along three axes. Most neural network models position points in two coordinates (*X* and *Y*) due to the complexity of depth estimation when using a camera. A number of algorithms (for example, MediaPipe) simulate the determination of the $pz_{i}$ coordinate relative to some reference point, but this value is inaccurate.

Therefore, to accurately determine the position of a person in space along all three axes using several (at least two) cameras, the following is calculated:

(27) $$\begin{array}{c} px_{i}=cx_{1,i}/kx,\\ py_{i}=\left(cy_{\max}-0.5\left(cy_{1,i}+cy_{2,i}\right)\right)/ky,\\ pz_{i}=\left(cx_{\max}-cx_{2,i}\right)/kx, \end{array}$$

where $cx_{1,i}$ and $cx_{2,i}$ are the position of point $p_{i}$ on the first and second cameras, respectively, along the *X* axis;

$cy_{1,i}$ and $cy_{2,i}$ are the position of point $p_{i}$ on the first and second cameras, respectively, along the *Y* axis;

$cx_{\max}$ and $cy_{\max}$ are the maximum pixel values along the *X* and *Y* axes, respectively;

kx and ky are coefficients for converting pixels into meters, determined by taking into account the length of the limbs and correlating them with the length of the corresponding recognized segment on the frame.

As a result of the transformation, the target point is formed as follows (27):

(28) $$tp_{i}=\langle px_{i},py_{i},pz_{i}\rangle ,$$

The resulting target point format corresponds to Formula (6).

Another approach is to use triangulation, the process of determining a point in three-dimensional space given its projections onto two or more images. To calculate the coordinates of a point in three-dimensional space, it is necessary to know the coordinates of its projections on images and the projective matrices of cameras [48]. The projective matrix MP of a certain camera can be represented as a combination of the matrices MA (containing the internal parameters of the camera) and MR (rotation), as well as the displacement vector of the VT, which describe the change in coordinates from the world coordinate system to the coordinate system relative to the camera:

(29) $$MP=MA[MR|VT]=\left[\begin{array}{ccc} f_{x} & 0 & c_{x}\\ 0 & f_{y} & c_{y}\\ 0 & 0 & 1 \end{array}\right]\left[\begin{array}{cccc} \begin{array}{l} mr_{11}\\ mr_{21}\\ mr_{31} \end{array} & \begin{array}{l} mr_{12}\\ mr_{22}\\ mr_{32} \end{array} & \begin{array}{l} mr_{13}\\ mr_{23}\\ mr_{33} \end{array} & \begin{array}{l} vt_{1}\\ vt_{2}\\ vt_{3} \end{array} \end{array}\right]$$

where $\left(c_{x},c_{y}\right)$: the coordinates of the camera central point;

$\left(f_{x},f_{y}\right)$: focal length in pixels.

At the base of the three-dimensional reconstruction of the object points by the values of point projection positions on the images from all cameras is the epipolar geometry. It provides a condition for searching for pairs of corresponding points on two images: if it is known that the point x on the plane of the first image corresponds to the point $x^{\prime }$ on the plane of the other image, then its projection must lie on the corresponding epipolar line. According to this condition, for all corresponding pairs of points $x↔x^{\prime }$, the following relation is true:

(30) $$x^{\prime }MFx=0$$

where MF is a fundamental matrix of size 3×3 and rank equal to two.

For some point X, given in three-dimensional space, the following projection formula is valid, expressed in homogeneous coordinates:

(31) $$x_{i}=MP_{i}X,$$

where $x_{i}=w{\left(u_{i},v_{i},1\right)}^{T}$ are the homogeneous coordinates of some point on the plane of the i-th image (obtained from the i-th camera during the second stage), including the position on the image of $u_{i}$ (along the *X* axis) and $v_{i}$ (along the *Y* axis);

w: the scaling factor;

$MP_{i}$: the projection matrix of the i-th camera obtained earlier.

To simplify calculations, the projection matrix of a camera is often represented in the following form:

(32) $$MP_{i}=\left[\begin{array}{c} mp_{i}^{1T}\\ mp_{i}^{2T}\\ mp_{i}^{3T} \end{array}\right]\left(MP_{i}\in {\mathbb{R}}^{3\times 4}\right),$$

where $mp_{i}^{jT}$ is the j-th row of the matrix $MP_{i}$.

Therefore, Equation (31) can be represented as follows:

(33) $$\begin{array}{c} wu_{i}=mp_{i}^{1T}X,\\ wv_{i}=mp_{i}^{2T}X,\\ w=mp_{i}^{3T}X. \end{array}$$

The following system of equations is obtained by considering w as a scaling factor:

(34) $$\begin{array}{l} u_{i}mp_{i}^{3T}X-mp_{i}^{1T}X=0,\\ u_{i}mp_{i}^{3T}X-mp_{i}^{2T}X=0. \end{array}$$

Since X is a homogeneous representation of coordinates in three-dimensional space, to calculate them, it is necessary to obtain $x_{i}$ and $MP_{i}$ for at least two cameras. To solve the system of Equation (34), there are a large number of implemented triangulation algorithms, for example, L2, direct linear transform, or other approaches implemented in the OpenCV computer vision library [49]. As a result of their work, a vector of three-dimensional coordinates similar to (28) is also formed.

If, during the exercise, it is necessary to track the movement of the target point only along two coordinates, then it is possible to use Formula (27), without taking into account the second camera and the need to calculate values along the third coordinate axis. An important point of this simplification is that it is necessary to correlate the normalized length of a body segment, obtained from the recognition algorithm (for example, MediaPipe) and expressed in pixels, with the real size of this segment, defined in meters. Using this ratio allows the calculation of all body segments in metric values.

### 2.3. Application of Machine Learning Algorithms for Analysis and Classification of Musculoskeletal Rehabilitation Exercises

Theoretical studies described in Section 2.1 and Section 2.2 on modeling the processes of monitoring and evaluating musculoskeletal rehabilitation exercises and algorithms for processing information from various sources make it possible to collect, analyze, and evaluate user actions in the rehabilitation process. However, problem (16) remains open; that is, it is necessary to determine a machine learning algorithm that allows classifying of user actions or movements as a kind of exercise. The importance of this task lies in the fact that, without an automated determination of the current exercise, it is impossible to correctly assess the quality of its performance since each exercise has its own trajectory of movements and spatio-temporal characteristics.

In Section 1.2, the main approaches and existing research in the field of the machine learning application in the rehabilitation and tracking of human movements are reviewed. Based on the presented model and algorithms, the procedure for applying machine learning technologies for the analysis and classification of musculoskeletal rehabilitation exercises is described.

For each exercise $e_{k}\in E$, a trajectory of the target points movement $TP_{k}$ is specified, including tuples of three-dimensional coordinates of one or more points, the number of which is specified by the variable m. Thus, the total dimension of the exercise initial data has the following form:

(35) $$\dim(TP_{k})=m\times N\times 3,$$

where N is the number of recorded sets of target points, corresponding to the size of the set $\left|TP_{k,m}\right|$, taking into account the supposition that, within $TP_{k}$, the lengths of the trajectories of all target points are equal.

To approximate (16), it is necessary to analyze the dynamics of changes in the position of the target points, and the use of measurement at one point in time does not allow one to determine the exercise since a person can occupy similar positions while performing various exercises. On the other hand, using the entire dataset $TP_{k}$ leads to the problem of a lack of a single dimension for all exercises due to their different durations (N).

To solve this problem, the classical approach is to determine the size Q of some window W, which selects a fixed-length fragment from the input data sequence. Such fragments of the same size are processed by machine learning algorithms with some shift (step) S until the window extracts the last fragment. This allows one to process time sequences of any length and create a forecast for each of them (in the framework of this study, an exercise category for each fragment). For the original time sequence, the resulting output is obtained:

(36) $$\begin{array}{c} \dim(TP_{k})=N_{W}\times m\times Q\times 3,\\ N_{W}=(N-Q)/S+1, \end{array}$$

where $N_{W}$ is the number of fragments determined on the basis of the following calculation; (N−Q) is the number of elements that are used to form complete fragments. Division by S shows how many such complete fragments can exist. The unit in expression (36) determines the possibility of adding the last incomplete fragment, which can be shorter than Q if N is not exactly divisible by Q (in this case, the last fragment consists of the last Q values).

Using expression (36), the entire initial time sequence is processed. Then, the machine learning algorithm required to approximate the expression (16) takes as input a multidimensional vector X of the format $\left(\left|TP\right|\times N_{W},m,Q,3\right)$ and at the output returns a vector Y belonging to a certain category of exercise with size |C|. The mapping X→Y is specified on the entire set of fragments of target point trajectories; even if the fragment initially does not have an exercise category $c_{q}\in C$, it can be assigned a new category $c_{u}\in C$, to which all unrecognized fragments are assigned [50].

Next, it is necessary to determine the optimal machine learning algorithm for solving the classification problem. Since multidimensional time sequences are processed, the following algorithms and architectures are chosen as possible solutions:

- DecisionTreeClassifier: decision trees for multiclass classification; the input of the algorithm must be an array of the format (the number of examples, the number of features), which requires transformation:

(37) $$(\left|TP\right|\times N_{W},m,Q,3)\to (\left|TP\right|\times N_{W},m\times Q\times 3);$$

- KNeighborsClassifier: k-nearest neighbor classifier; the input data format is identical to decision trees and requires transformation (37);
- RandomForestClassifier: a meta estimator that trains a set of decision trees; input data format needs to be converted (37);
- NN: multilayer neural networks including dense layers;
- LSTM: multilayer recurrent neural networks, including layers of long-term memory;
- CNN: convolutional neural networks using 1D convolutional layers (Conv1D) to identify and generalize features in a time sequence;
- CNN + Transformer: a combined neural network that first identifies the main features of the data using convolutional layers, then uses the MultiHeadAttention layers to extract from the set all the most important features for the current class. As a basis for the architecture of this network, it is proposed to use MobileViT [51], which requires a transformation of the input data to the following form:

(38) $$(\left|TP\right|\times N_{W},m,Q,3)\to (\left|TP\right|\times N_{W},m,Q,3,1);$$

The presented five architectures of machine learning algorithms make it possible both to identify the best solution in the process of selecting the hyperparameters of each of the architectures (tree depth for the first two algorithms, the number of layers and neurons for the rest), and to determine their applicability for analyzing data on the process of human movement.

## 3. Results

### 3.1. Experimental Research Design

Experimental studies had the structure described below. A small control group was formed which performed the following exercises:

Task 1: raise the arm to a level parallel to the floor, then lower it (10 repetitions). The target point is the wrist of the hand performing the exercise $e_{1}$ by the trajectory $TP_{1}=\{tp_{0},tp_{1},\ldots ,tp_{N}\}$, where each trajectory point $tp_{i}$ corresponds to coordinates according to Formula (6).

$$\mathrm{Reference}\;\mathrm{values}:\;x_{1}^{\min}=x_{0},x_{1}^{\max}=x_{0}+x_{hand},y_{1}^{\min}=y_{0},y_{1}^{\max}=y_{0}+y_{hand},$$

where $\langle x_{0},y_{0}\rangle$ are the initial coordinates of the target point (hand) at the beginning of the exercise; $\langle x_{hand},y_{hand}\rangle$ is the final position of the subject’s hand in a state parallel to the floor. Thus, the hand must pass the distances $x_{hand}$ and $y_{hand}$ along the corresponding axes.

Task 2: lifting the leg to the level of the step, which is imitated by stepping onto the box and then returning to the starting position (10 repetitions). The target point is the foot of the leg performing the exercise $e_{2}$ by the trajectory $TP_{2}$.

$$\mathrm{Reference}\;\mathrm{values}:\;x_{2}^{\min}=x_{0},x_{2}^{\max}=x_{0}+x_{foot},y_{2}^{\min}=y_{0},y_{2}^{\max}=y_{0}+y_{foot},$$

where $\langle x_{0},y_{0}\rangle$ are the initial coordinates of the leg at the beginning of the exercise;$\langle x_{foot},y_{foot}\rangle$ is the final position of the subject’s leg while on the box (the same for all participants).

Task 3: standing up from a chair and then returning to the starting position. (10 repetitions). Target points are lumbar region and neck, along which the corresponding trajectories $TP_{3,1}=\{tp_{0,1},tp_{1,1},\ldots ,tp_{N,1}\}$ and $TP_{3,2}=\{tp_{0,2},tp_{1,2},\ldots ,tp_{N,2}\}$ are tracked in exercise $e_{3}$.

$$\mathrm{Reference}\;\mathrm{values}\;\mathrm{for}\;\mathrm{belt}:\;y_{3,1}^{\min}=y_{0,1},\;y_{3,1}^{\max}=y_{body};\;\mathrm{for}\;\mathrm{neck}:\;y_{3,2}^{\min}=y_{0,2},\;y_{3,2}^{\max}=(y_{0,2}-y_{0,1})+y_{body},$$

where $\langle x_{0,1},y_{0,1}\rangle$ is the initial position of the lumbar region; $\langle x_{0,2},y_{0,2}\rangle$ is the initial position of the neck; $\langle x_{body},y_{body}\rangle$ is the position of the subject’s lumbar region in a standing position.

The experiment was carried out as follows: the subject with the tracking system hardware connected to him/her was in the starting position, and the required lengths of his body parts (arms, legs, and torso) were measured, which was used later when working with systems based on normalized coordinates. Next, the exercise was performed for 10 repetitions. The values of the target points were fixed in their extreme positions, and the trajectories of their movements were also recorded. Further, the collected data were used to evaluate the accuracy of tracking the target points and to classify the exercises performed.

During the experiment, data were collected from five motion tracking systems:

S1: Android mobile phone with built-in inertial navigation system and 100 Hz recording frequency.

S2: Wireless inertial navigation system based on the MPU-9250 MotionTracking device, recording frequency: 500 Hz.

S3: HTC Vive Tracker sensor set, recording frequency: 60–100 Hz.

S4: Motion capture suit Perception Neuron with 32 sensors, animation frequency: 60 Hz.

S5: MediaPipe Pose computer vision system and 1080 p 30 Hz cameras.

Each one of the listed tracking systems was fixed to the human body at the target point, after which data were collected when performing the exercise in the required number of repetitions. The total weight of the tracking systems does not exceed 1.5 kg, distributed mostly evenly, since the largest contribution is made by the Perception Neuron suit (1.1 kg). The collected data were digitized in accordance with the algorithms in Section 2.2. Since each participant’s height and limb length were known, it was possible to determine the optimal position of the target point, relative to which the *MSE* and metrics based on the Euclidean distance were calculated. A comparison of systems by metrics was carried out using statistical analysis according to the Kruskal–Wallis method to determine if there was a statistically significant difference between the medians of three or more independent groups. The Kruskal–Wallis test did not assume normality of the data and was much less sensitive to outliers than a one-way ANOVA.

**Figure 1:**
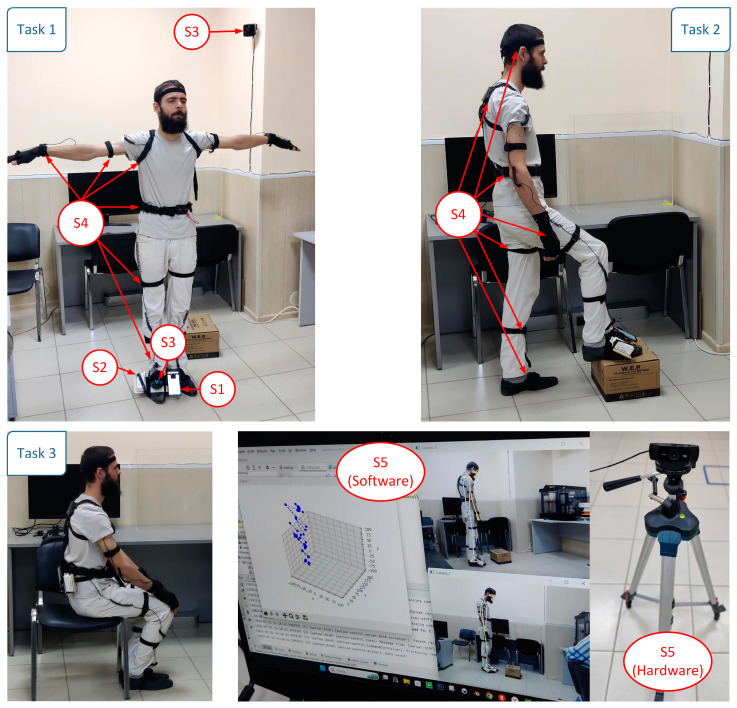
Fragments of experimental studies.

Part of the experiment is shown in Figure 1.

As part of the experiment, the quality of the exercise was not evaluated in terms of position, speed, or trajectory since these parameters were set individually by the attending physician. However, these metrics were used to compare tracking systems. For each exercise and repetition, the minimum and maximum positions of the target point along the three axes were analyzed, after which the distance traveled by the point was calculated. Additionally, the deviation of the target point from the initial and final positions was estimated.

The selected control group of eight people for the correct comparison of tracking systems does not have diseases of the musculoskeletal system. All participants were informed about the conditions of the experiment and agreed to participate in it.

As a result, the most accurate tracking system was selected, taking into account the requirements for the comfort of its use in conditions of musculoskeletal rehabilitation. Further, this tracking system was used in the second experiment when comparing different machine learning algorithms in order to solve the problem of classifying musculoskeletal rehabilitation exercises. To ensure the correct operation of machine learning algorithms on all tracking systems, the recording frequency was aligned to a common value of 30 Hz.

### 3.2. Comparison of Motion Tracking Systems in Musculoskeletal Rehabilitation Exercises

At the first stage of the experimental studies, each of the participants in the control group performed the three exercises discussed above (10 repetitions each). Each measurement was processed according to the algorithms in Section 2.2. The experiment was conducted in a closed room with a free space of 2.5 × 1.8 m with the same equipment for each participant (Figure 1). At the beginning of each experiment, the sensors were calibrated to reduce the influence of electromagnetic interference. In the experimental area, all equipment not used directly in the study was turned off. The duration of one repetition of the exercises was as follows: for Task 1—7.1 ± 1.7 s, for Task 2—6.3 ± 1.9 s, for Task 3—6.1 ± 1.3 s.

The collected and processed data were filtered to eliminate errors caused by electromagnetic interference, loss of sensors, or the accumulation of a too-large error in INS. The recording considered a result an error if the distance traveled by the target point exceeded 3 m. As a result, 1335 exercise records were collected. Table 1 shows the amount of data collected for each exercise and tracking system. It was assumed that the target points of exercise 3 were considered separately; for the upper point, we denoted this as Task 3 (TP), and for the lower point, Task 3 (BP). The last line of the table reflects the percentage of correct entries from the maximum possible number, which leads us to conclude that the recording and tracking systems are stable.

Table 2 shows the maximum values in each of the axes for the target point ($x_{k}^{\max}$, $y_{k}^{\max}$, and $z_{k}^{\max}$). After that, the distances traveled by the target points along the corresponding axes ($x_{k}^{path}$, $y_{k}^{path}$, and $z_{k}^{path}$) were calculated, as were two metrics, *MSE* and D between the positions of the target point and its reference position, calculated on the basis of the overall body characteristics for each participant, as well as given constants (box and chair sizes). The metrics were calculated both for the final position and for the distance traveled (since some systems saved point values in normalized coordinates, the starting point did not always have zero values on all axes, or the direction of movement after calibration was not always in the positive direction). The lowest metric values for each exercise are shown in bold. The last column is the p-value of the Kruskal test for assessing the statistical significance of the sample equality among themselves. If the p-value < 0.05, then the hypothesis about the equality of the samples is refuted and there is a statistical difference between them. The obtained p-value indicates that the samples are completely distinct from one another.

In the course of the obtained experimental data analysis, it can be concluded that target point tracking is the most accurate when using virtual reality trackers (S3). Next in accuracy are the motion capture suit (S4) and computer vision (S5). The systems based on accelerometers (S1 and S2) provide the least accuracy. It should be noted that in a number of scenarios, the S4 and S5 systems show the best results. By evaluating the average results for all exercises, it can be concluded that the S5 system is superior to S4.

The results in Table 2 are consistent with existing studies. INS, especially those based on mobile sensors, tend to accumulate errors, which can lead to an absolute positioning error of 0.15 to 0.8 m [52,53]. In [54], phone sensors were used; when comparing different approaches, the error was up to 5% when moving over long distances (hundreds of meters), but with small movements, as in our study, it can be higher. High accuracy (up to 0.07 m) when using an IMU can be achieved by combining several devices connected to a single network [55], but such a design could approach motion capture systems in terms of implementation complexity, yielding to them in universality.

In [56], when using six virtual reality trackers, an average deviation of 0.02 m was obtained. Motion capture systems, similarly to inertial systems, are susceptible to error accumulation and, depending on the movements performed, can show different accuracy. The values obtained in the experiment are comparable with existing results of 0.2 m and an average deviation of up to 0.41 m [57].

Computer vision systems are often evaluated by angle deviations; however, a number of studies give an error estimate from 0.02 to 0.16 m [58], and a global comparative study [59] gives an error estimate from several to tens of centimeters (depending on the task and method). The results obtained in Table 2 are fully consistent with the current limitations of computer vision technology.

### 3.3. Assessment of the Classification Accuracy of Musculoskeletal Rehabilitation Exercises

The next stage of research is to analyze the possibility of classifying musculoskeletal rehabilitation exercises performed by a person using various machine learning algorithms. The list of compared models is presented in Section 2.3. The final parameters of the models that provide the best classification performance are presented in Table 3.

**Table 3:** Parameters of selected machine learning algorithms.

Model	Description
DecisionTreeClassifier (DT)	Standard decision tree regressor with max_depth = 10
KNeighborsClassifier (KNN)	Standard classifier based on k-nearest neighbors
RandomForestClassifier (RF)	Standard random forest with n_estimators = 20, max_depth = 10
Simple neural network (NN)	Multilayer neural network with 4 hidden Dense layers of 200 neurons with ReLU activation function, 1 Dropout layer (20% dropout rate)
Long short-term memory neural network (LSTM)	Multilayer neural network with two LSTM layers (20 and 50 neurons), 2 hidden Dense layers of 100 neurons, 1 Dropout layer (20% dropout rate)
Multiple neural network (CNN)	Multilayer Neural Network with 4 Blocks from the Conv1D Convolutional Layer (number of filters from 32 to 256, convolutional kernel = 3) combined with BatchNormalization, followed by GlobalAvgPool1D and 1 Dense layer of 100 neurons
CNN + Transformer (Transformer)	A model based on the MobileViT architecture shown in Figure 2

**Figure 2:**
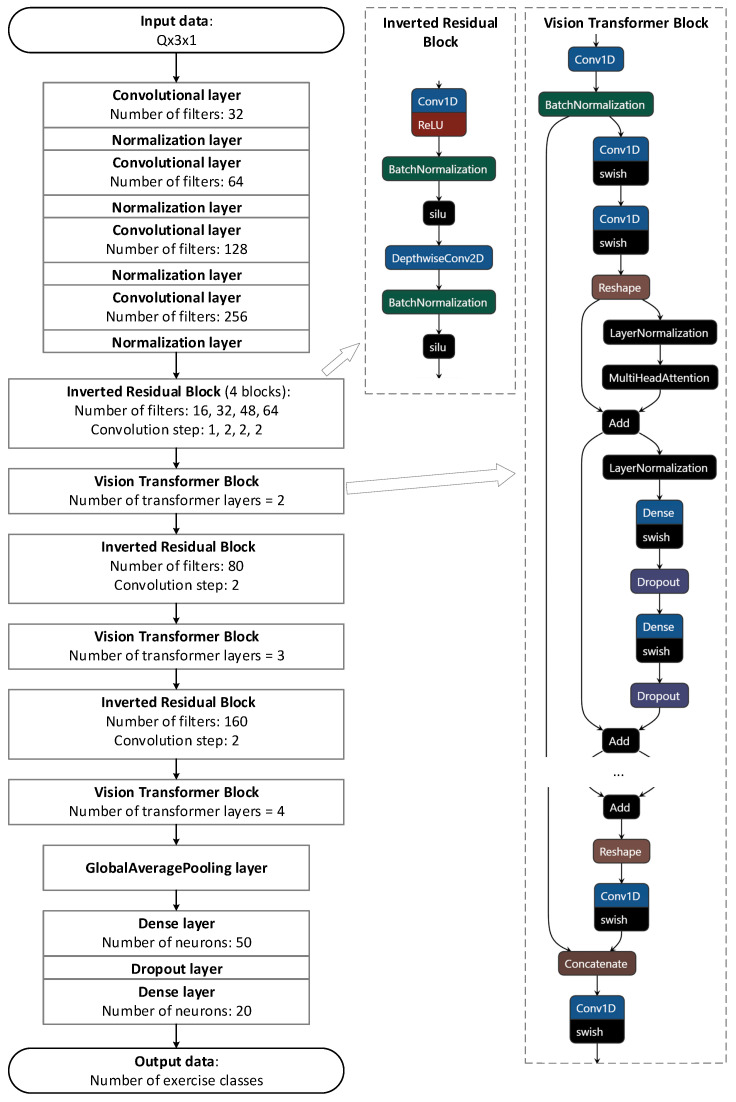
Upgraded MobileViT architecture of the Transformer model.

It should be noted that various tracking systems allow the extraction of information not only around the target point but also around other points of the human body (for example, systems S4 and S5) in the standard mode of operation, while other systems require the addition of more sensors, which briefly complicates the process of collecting and processing data.

At the next stage of research, experiments were carried out to determine the optimal size of the analyzed fragment Q. For this, data from all tracking systems were used, after which all selected models were trained for 10 epochs on the generated datasets at Q = 10, 25, 50, 100, 200. The results of this comparison are presented in Table 4. The best results for each model are highlighted in bold font. The table also shows the dimensions of the dataset obtained for each Q.

When choosing the size of Q, an important aspect must be taken into account: a too-long window W does not allow the analysis of short exercises and increases the delay in real-time analysis. On the other hand, it is obvious that too-short fragments do not allow one to reveal the characteristic features of the exercises. In addition, for very large Q, if the original record has a length less than Q, it is not included in the dataset. Based on the data in Table 4, this happens for Q>100. At a value of Q<100, the accuracy of the trained models is low. Given the above, the optimal value of Q is 100, since this allows one to take all the data into account and ensure good accuracy.

**Figure 3:**
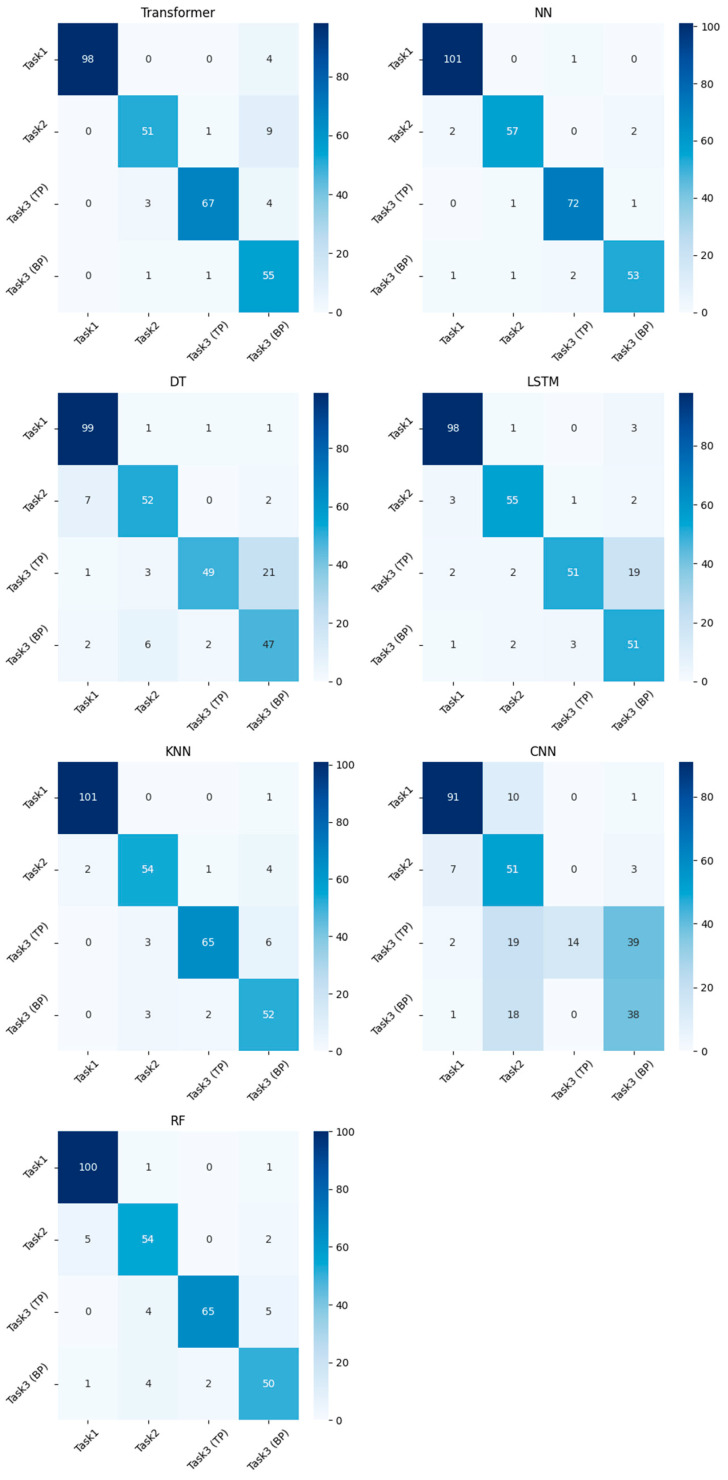
Classification error matrices of different models (dataset of systems S3–S5).

Thus, the collected data were processed with a step S = 50 and a window size Q=100, which made it possible to form a dataset of 1966 values. In total, 80% of the data was used for training and 20% for testing. The data were also divided into samples related to each type of tracking system. Also, in the course of the previous experiment, the undertraining of the models was clearly visible; therefore, further training of neural networks was carried out for 50 epochs. Table 5 presents the results of comparing models for each of the tracking systems (by accuracy metric), as well as for the S3–S5 dataset (last column). Figure 3 shows the error matrices for all models trained on the S3–S5 dataset. The grouping of data S3–S5 was chosen because of its greater accuracy relative to systems S1 and S2.

When training neural networks, it was assumed that the upper and lower target points in exercise 3 were considered separately since the difference in the number of points at the input of the models would automatically reveal exercise 3 relative to the others. On the other hand, such a division is of interest in terms of the ability of models to determine the exercise even for one target point out of two.

An analysis of the results obtained suggests that the NN model shows the highest accuracy on the dataset used, followed by RF and KNN. They are stable both on individual datasets and on a complete set that combines information from various sources. It should be noted the simplicity of these models and the speed of their learning, which clearly indicate their applicability to real problems. The Transformer model also performs well on data from S3–S5 systems but has high complexity and a long training time, like LSTM.

Due to the low accuracy of tracking target points on systems S1 and S2, there is a low accuracy of the models on the data from these systems. The training of S3–S5 systems demonstrates very high accuracy results, which allows us to consider their applicability.

An experiment was also conducted to use all 33 points of the S5 computer vision system to classify exercises. Since all points of the human body were analyzed, it is possible to classify exercise 3 in the same way as the rest without the need to separate into upper and lower target points. The results are presented in Table 6. The best result was shown by the NN, RF, KNN, and Transformer models, which is consistent with previous experiments. The table also presents the values of the F1-measure metric for each of the classes.

The results presented in Table 6 indicate that the use of all points of the body affects the solution of the problem of the classification of musculoskeletal rehabilitation exercises. It should be noted that a number of models handle the increased volume of input data rather poorly. Other models (for example, Transformer), on the other hand, carry out classification at a high level.

Next, it is important to provide a summary of the findings from the investigations.

## 4. Discussion

The conducted experimental studies made it possible not only to quantitatively compare different tracking systems in terms of the accuracy of positioning human target points but also to draw conclusions about the qualitative differences between them when solving problems of musculoskeletal rehabilitation. We consider these features of motion tracking systems in the process of confirming or refuting the hypotheses put forward earlier.

To confirm the first hypothesis, the issue of ranking tracking systems by accuracy was considered. Indeed, when performing different types of exercises and recording the positions of the target points using capture systems, significantly different results were obtained. The empirical investigations reveal that the S3 system utilizes virtual reality trackers, which have design features that ensure the highest level of accuracy. The disadvantage of the S3 system is that there are problems when the sensor is covered by clothing or the body, which leads to incorrect values. It is also necessary to take into account the need to install base stations and the high cost of this system, as it should include a VR headset that is not directly involved in the process. For those systems where virtual reality is integrated into the rehabilitation procedure, this disadvantage is less important.

Next in precision are systems based on motion capture suits (S4) and the use of cameras with computer vision (S5). In a number of scenarios, comparable results were shown by these systems, but based on the results, the superiority of the S5 system can be discussed. Its advantages include the absence of additional sensors and ease of operation since it is only required to place a person in the tracking zone and periodically calibrate the cameras for the operation of triangulation algorithms. In addition, it is possible to build an S5 system based on a single camera (when tracking target points in two-dimensional space), which is discussed in Section 2.2.4. One of the important disadvantages of the S4 system is that data recording is not always correct due to external interference or the accumulation of sensor errors (about 10% of the records were eliminated after preprocessing; see Table 1).

Systems S1 and S2, based on inertial sensors (gyroscope, accelerometer), allow one to determine the movement of the target point autonomously. S1 is also extremely accessible to the general population. On the other hand, the positioning accuracy due to the accumulation of errors, noise, and sensor errors is quite high. It should be noted that from 36 to 32% of the records were unsuitable for processing due to high discrepancies with real data and the impossibility of restoring the correct movement trajectory from the initial acceleration values. This does not allow one to consider the effective use of such systems in the organization of the musculoskeletal rehabilitation process.

Hypothesis 1 is completely confirmed. In the process of its confirmation, it was found that systems S3, S5, and S4 have the best accuracy. S1 and S2 are not recommended for use.

Given the specifics of the subject area—performing musculoskeletal rehabilitation exercises—accuracy is not the only criterion for choosing a tracking system. Therefore, in the process of testing hypothesis 2 about the limitations of using the considered tracking systems and monitoring the progress of the exercises (Figure 1), the following conclusions were drawn:

- The use of a motion capture suit for people with diseases of the musculoskeletal system is impossible, as it requires attaching too many sensors and equipment to them;
- Systems S1 and S2 require fixing the corresponding devices on the human body, which is not possible or convenient for all points, especially in the case of S2. In addition, these systems, even within the framework of the experiments performed, are extremely inconvenient when collecting more than one target point (since this requires a synchronization system for launching and processing data);
- During the operation of the S3 system, there are similar disadvantages, to which is added the need to install base stations indoors, in the area where the VR helmet should also be located; this greatly limits the use of these systems in both outpatient and inpatient environments;
- The S5 system does not contact the user directly, which does not impose any restrictions on him/her; cameras can be fixed at an arbitrary distance and track all points of the human body at the same time; the disadvantages include possible system failures if other people appear in the visibility zone, but this aspect can be eliminated by additional software that takes into account the appearance of the current user.

Thus, hypothesis 2 is also confirmed. In addition to differences in tracking accuracy, the considered systems have a number of limitations that make it difficult to use them in outpatient and inpatient environments. Among all systems, therefore, preference should be given to the S5 system because it does not affect the user and is quite accurate. In addition, the use of all points of the human body allows one to classify exercises with the highest accuracy (Table 6).

To confirm hypothesis 3, the corresponding studies were carried out, as presented in Section 3.3. A comparison was made of various machine learning algorithms on each of the tracking systems under consideration, and a search was conducted for a universal model that can process and classify data from any source. In the course of these experiments, the optimal window size for analyzing movement data was determined, and models (NN, RF, and KNN) were selected that provide an accuracy of exercise classification up to 96%. The use of all points of the human body in combination with the computer vision system (S5) allows one to obtain a classification accuracy of 100% on NN, RF, KNN, and Transformer models.

Thus, the third hypothesis is also confirmed: the machine learning models trained on the collected dataset make it possible to determine the performed musculoskeletal rehabilitation exercise with high accuracy.

## 5. Conclusions

This study considers the task of tracking a person in the process of musculoskeletal rehabilitation using various motion capture systems in order to ensure the highest positioning accuracy. In the course of this study, the following tasks were successfully solved.

An analysis of the subject area was carried out, on the basis of which the modeling of the processes of monitoring and evaluating musculoskeletal rehabilitation exercises was performed, including a description of the procedures for determining the position of the user’s body parts, the amplitude and speed of their movement, the current exercise, and the quality of its implementation. Algorithms for processing data from various motion tracking systems have been developed.

In the course of experimental studies, based on the developed models and algorithms, data were collected from various user tracking systems. They were compared and ranked in terms of accuracy: the best results were shown by a system based on virtual reality trackers, followed by a computer vision system and a motion capture suit; the worst results were obtained using INS. In the course of the experiments, limitations were identified for the use of motion tracking systems when performing musculoskeletal rehabilitation exercises.

Various machine learning models were developed and trained to solve the problem of classification of musculoskeletal rehabilitation exercises, and their comparison was carried out. It is found that fairly simple models show the best results: a multilayer dense neural network model, a random forest, and a classifier based on k-nearest neighbors. An experiment was also conducted in the classification of exercises with the processing of all points of the body received from the computer vision system. The NN, KNN, RF, and Transformer models showed good results, which further confirms the applicability of this tracking system.

The hypotheses put forward at the beginning of this study about the ranking of tracking systems according to the positioning accuracy of human target points, about the presence of restrictions on their use in the field of musculoskeletal rehabilitation, and about the possibility of classifying musculoskeletal rehabilitation exercises are fully confirmed.

Thus, the main contribution of this study lies in the following aspects:

- A comparative analysis of human movement tracking systems adapted for musculoskeletal rehabilitation, which revealed that systems based on computer vision are most preferable in this area;
- Modeling the process of monitoring and evaluating specific exercises for an adapted tracking system, taking into account restrictions on low mobility of users (use in outpatient and inpatient environment, lack of sensors directly on the human body);
- Development and testing of data processing algorithms for selected human tracking systems, which allows for each of them to determine the qualitative aspects of musculoskeletal rehabilitation exercises, and monitor and evaluate them;
- Implementation of exercise classification algorithms with the ability to automatically determine exercises based on developed and trained machine learning models, which can also be used to identify human movement patterns and recognize types of activity for the subsequent implementation of automatic systems for monitoring and tracking human activities.

The aim of further research is to test and deepen the results obtained: expanding the range of classified exercises using the selected tracking system based on computer vision; integration of trained machine learning models for the development of software for monitoring and evaluating musculoskeletal rehabilitation exercises, operating on the basis of computer vision technologies. It is also planned to use the developed algorithms and tools in assessing changes in a person’s condition during the use of musculoskeletal rehabilitation systems.

## Figures and Tables

**Table 1:** The amount of data collected from each tracking system.

Exercise	S1	S2	S3	S4	S5
Task 1	49	46	80	73	71
Task 2	60	68	79	70	76
Task 3 (TP)	49	42	79	73	80
Task 3 (BP)	61	48	78	73	80
Percentage of correct data	68%	64%	99%	90%	96%

**Table 2:** Comparison of tracking systems for accuracy.

Exercise	Metrics	S1	S2	S3	S4	S5	p-Value
Task 1	$x_{k}^{\max}$	0.16 ± 0.26	0.24 ± 0.45	0.41 ± 0.07	0.3 ± 0.06	0.06 ± 0.07	0.0
	$y_{k}^{\max}$	0.26 ± 0.47	0.38 ± 0.6	0.71 ± 0.06	0.41 ± 0.07	0.61 ± 0.29	0.0
	$z_{k}^{\max}$	0.11 ± 0.17	0.31 ± 0.7	0.23 ± 0.04	0.05 ± 0.05	0.09 ± 0.1	0.0
	$x_{k}^{path}$	0.27 ± 0.27	0.34 ± 0.47	0.44 ± 0.07	0.31 ± 0.06	0.48 ± 0.22	0.0
	$y_{k}^{path}$	0.61 ± 0.48	0.48 ± 0.65	0.72 ± 0.06	0.42 ± 0.06	0.63 ± 0.26	0.0
	$z_{k}^{path}$	0.3 ± 0.25	0.4 ± 0.69	0.24 ± 0.04	0.09 ± 0.06	0.26 ± 0.09	0.0
	S	0.15 ± 0.09	0.17 ± 0.2	0.23 ± 0.05	0.16 ± 0.06	0.63 ± 0.36	0.0
	*MSE*	0.84 ± 0.37	0.96 ± 0.37	**0.11 ± 0.05**	0.31 ± 0.13	0.58 ± 0.17	0.0
	D	0.89 ± 0.23	0.96 ± 0.2	**0.32 ± 0.07**	0.55 ± 0.09	0.75 ± 0.11	0.0
	*MSE* (path)	0.53 ± 0.26	0.89 ± 0.38	**0.1 ± 0.04**	0.3 ± 0.12	0.2 ± 0.26	0.0
	D (path)	0.71 ± 0.19	0.92 ± 0.2	**0.3 ± 0.07**	0.54 ± 0.09	0.36 ± 0.26	0.0
Task 2	$x_{k}^{\max}$	0.19 ± 0.37	0.14 ± 0.2	0.02 ± 0.02	0.25 ± 0.1	0.21 ± 0.15	0.0
	$y_{k}^{\max}$	0.08 ± 0.23	0.26 ± 0.29	0.3 ± 0.07	0.15 ± 0.06	0.15 ± 0.09	0.0
	$z_{k}^{\max}$	0.09 ± 0.18	0.26 ± 0.17	0.01 ± 0.02	0.07 ± 0.04	0.28 ± 0.22	0.0
	$x_{k}^{path}$	0.24 ± 0.37	0.31 ± 0.38	0.36 ± 0.06	0.28 ± 0.1	0.4 ± 0.24	0.0
	$y_{k}^{path}$	0.31 ± 0.41	0.48 ± 0.42	0.3 ± 0.07	0.15 ± 0.06	0.21 ± 0.13	0.0
	$z_{k}^{path}$	0.19 ± 0.24	0.36 ± 0.19	0.2 ± 0.05	0.11 ± 0.05	0.33 ± 0.2	0.0
	S	0.06 ± 0.03	0.23 ± 0.1	0.18 ± 0.04	0.16 ± 0.06	0.43 ± 0.29	0.0
	*MSE*	0.24 ± 0.42	0.17 ± 0.42	0.12 ± 0.02	**0.03 ± 0.04**	0.06 ± 0.07	0.0
	D	0.42 ± 0.25	0.34 ± 0.23	0.34 ± 0.03	**0.16 ± 0.09**	0.21 ± 0.14	0.0
	*MSE* (path)	0.32 ± 0.78	0.37 ± 1.27	**0.01 ± 0.02**	0.03 ± 0.04	0.08 ± 0.21	0.0
	D (path)	0.41 ± 0.39	0.36 ± 0.49	**0.08 ± 0.07**	0.14 ± 0.09	0.2 ± 0.2	0.0
Task 3 (TP)	$x_{k}^{\max}$	0.14 ± 0.17	0.05 ± 0.07	0.02 ± 0.02	0.17 ± 0.05	0.38 ± 0.28	0.0
	$y_{k}^{\max}$	0.5 ± 0.64	0.17 ± 0.19	0.41 ± 0.02	0.06 ± 0.01	0.33 ± 0.22	0.0
	$z_{k}^{\max}$	0.5 ± 0.61	0.25 ± 0.31	0.01 ± 0.02	0.02 ± 0.02	0.46 ± 0.28	0.0
	$x_{k}^{path}$	0.23 ± 0.22	0.23 ± 0.46	0.44 ± 0.12	0.18 ± 0.06	0.45 ± 0.25	0.0
	$y_{k}^{path}$	0.76 ± 0.56	0.39 ± 0.39	0.45 ± 0.04	0.13 ± 0.07	0.47 ± 0.22	0.0
	$z_{k}^{path}$	0.68 ± 0.63	0.4 ± 0.31	0.29 ± 0.09	0.05 ± 0.02	0.52 ± 0.28	0.0
	S	0.2 ± 0.12	0.23 ± 0.17	0.27 ± 0.07	0.13 ± 0.05	0.67 ± 0.51	0.0
	*MSE*	0.61 ± 0.93	0.33 ± 0.1	0.23 ± 0.02	0.26 ± 0.06	**0.16 ± 0.19**	0.0
	D	0.69 ± 0.36	0.57 ± 0.1	0.48 ± 0.03	0.51 ± 0.06	**0.32 ± 0.23**	0.0
	*MSE* (path)	0.57 ± 1.05	0.46 ± 0.97	**0.03 ± 0.02**	0.21 ± 0.08	0.13 ± 0.16	0.0
	D (path)	0.63 ± 0.42	0.57 ± 0.38	**0.15 ± 0.07**	0.45 ± 0.1	0.3 ± 0.2	0.0
Task 3 (BP)	$x_{k}^{\max}$	0.17 ± 0.22	0.1 ± 0.11	0.01 ± 0.02	0.09 ± 0.02	0.21 ± 0.15	0.0
	$y_{k}^{\max}$	0.37 ± 0.37	0.16 ± 0.37	0.42 ± 0.03	0.07 ± 0.01	0.19 ± 0.13	0.0
	$z_{k}^{\max}$	0.13 ± 0.22	0.15 ± 0.12	0.01 ± 0.01	0.01 ± 0.02	0.29 ± 0.2	0.0
	$x_{k}^{path}$	0.3 ± 0.32	0.16 ± 0.17	0.36 ± 0.07	0.12 ± 0.03	0.29 ± 0.15	0.0
	$y_{k}^{path}$	0.61 ± 0.5	0.24 ± 0.37	0.43 ± 0.02	0.07 ± 0.01	0.25 ± 0.12	0.0
	$z_{k}^{path}$	0.49 ± 0.48	0.33 ± 0.56	0.24 ± 0.04	0.04 ± 0.02	0.34 ± 0.19	0.0
	S	0.16 ± 0.11	0.14 ± 0.13	0.2 ± 0.03	0.07 ± 0.02	0.35 ± 0.22	0.0
	*MSE*	0.32 ± 0.3	0.4 ± 0.45	0.24 ± 0.02	0.31 ± 0.06	**0.19 ± 0.18**	0.0
	D	0.52 ± 0.22	0.59 ± 0.23	0.49 ± 0.02	0.55 ± 0.05	**0.39 ± 0.21**	0.0
	*MSE* (path)	0.46 ± 0.97	0.33 ± 0.46	**0.03 ± 0.02**	0.29 ± 0.05	0.12 ± 0.15	0.0
	D (path)	0.56 ± 0.39	0.52 ± 0.24	**0.15 ± 0.07**	0.53 ± 0.05	0.29 ± 0.19	0.0
Mean	*MSE*	0.481 ± 0.59	0.434 ± 0.48	**0.175 ± 0.07**	0.23 ± 0.14	0.241 ± 0.25	
	D	0.614 ± 0.32	0.584 ± 0.3	**0.409 ± 0.09**	0.445 ± 0.18	0.411 ± 0.27	
	*MSE* (path)	0.463 ± 0.84	0.498 ± 0.93	**0.041 ± 0.04**	0.208 ± 0.13	0.131 ± 0.2	
	D (path)	0.566 ± 0.38	0.568 ± 0.42	**0.173 ± 0.11**	0.418 ± 0.18	0.286 ± 0.22	

**Table 4:** Comparison of selected machine learning models for different *Q*.

Model	*Q*					
	10	25	50	100	150	200
DT	61.84	62.42	66.25	71.32	**77.6**	74.36
KNN	77.34	77.51	78.84	81.73	**82.4**	76.92
RF	66.2	69.75	72.93	82.23	**84.8**	76.92
NN	58.31	59.72	63.54	69.04	**76.0**	71.79
LSTM	57.76	55.54	54.14	54.06	60.8	**64.1**
CNN	46.94	49.16	48.16	53.05	51.2	**56.41**
Transformer	50.86	54.84	**60.06**	54.06	24.8	35.9
Dataset size	(49,597, 10, 3)	(14,249, 30, 3)	(7184, 50, 3)	(1966, 100, 3)	(624, 150, 3)	(195, 200, 3)

**Table 5:** Comparison of machine learning algorithms.

Model	Data Source					
	S1	S2	S3	S4	S5	S3–S5 Systems
DT	58.97	50.82	84.55	88.89	**100.0**	81.29
KNN	69.23	63.93	92.73	**94.44**	**100.0**	92.52
RF	61.54	**70.49**	**94.09**	92.59	**100.0**	91.84
NN	**76.92**	59.02	**94.09**	**94.44**	95.24	**96.26**
LSTM	64.1	52.46	81.82	66.67	**100.0**	86.73
CNN	43.59	47.54	71.82	85.19	66.67	65.99
Transformer	46.15	29.51	92.27	90.74	**100.0**	92.18

**Table 6:** Comparison of machine learning algorithms at all points of the S5 system.

Model	F1-Measure by Classes			Accuracy
	Task 1	Task 2	Task 3	
DT	88.89	90.91	**100.0**	94.12
KNN	**100.0**	**100.0**	**100.0**	**100.0**
RF	**100.0**	**100.0**	**100.0**	**100.0**
NN	**100.0**	**100.0**	**100.0**	**100.0**
LSTM	45.45	0.0	0.0	29.41
CNN	90.91	75.0	93.33	88.24
Transformer	**100.0**	**100.0**	**100.0**	**100.0**

## Data Availability

Datasets available on request form corresponding author only as the data are sensitive and participants may be potentially identifiable.
